# The role of IR inactive mode in W(CO)_6_ polariton relaxation process

**DOI:** 10.1515/nanoph-2023-0589

**Published:** 2023-11-08

**Authors:** Oliver Hirschmann, Harsh H. Bhakta, Wei Xiong

**Affiliations:** Department of Chemistry and Biochemistry, University of California San Diego, La Jolla, CA 92093, USA; Materials Science and Engineering Program, University of California San Diego, La Jolla, CA 92093, USA; Department of Electrical and Computer Engineering, University of California San Diego, La Jolla, CA 92093, USA

**Keywords:** polariton, ultrafast dynamics, phonon assisted, vibrational strong coupling

## Abstract

Vibrational polaritons have shown potential in influencing chemical reactions, but the exact mechanism by which they impact vibrational energy redistribution, crucial for rational polariton chemistry design, remains unclear. In this work, we shed light on this aspect by revealing the role of solvent phonon modes in facilitating the energy relaxation process from the polaritons formed of a *T*_1*u*_ mode of W(CO)_6_ to an IR inactive *E*_*g*_ mode. Ultrafast dynamic measurements indicate that along with the direct relaxation to the dark *T*_1*u*_ modes, lower polaritons also transition to an intermediate state, which then subsequently relaxes to the *T*_1*u*_ mode. We reason that the intermediate state could correspond to the near-in-energy Raman active *E*_*g*_ mode, which is populated through a phonon scattering process. This proposed mechanism finds support in the observed dependence of the IR-inactive state’s population on the factors influencing phonon density of states, e.g., solvents. The significance of the Raman mode’s involvement emphasizes the importance of non-IR active modes in modifying chemical reactions and ultrafast molecular dynamics.

## 1 Introduction

Molecular vibrational polaritons (MVPs) represent a fascinating class of hybrid photon-matter states arising from the rapid energy exchange between molecular vibrations and optical cavity modes surpassing their individual dephasing lifetimes [1–3]. In the energy domain, this manifests when the coupling strength between the cavity and vibrational modes becomes greater than their individual linewidths, the so-called vibrational strong coupling (VSC) regime, resulting in the formation of two distinct bright eigenstates: the lower polariton (LP) and upper polariton (UP), separated in energy by Rabi splitting (Ω). Additionally, there also exists an ensemble of eigenstates of weakly coupled vibrational modes known as dark modes [2, 3]. Notably, vibrational polaritons exhibit a unique energy signature and encompass both photonic and molecular-like properties. In contrast, dark modes are solely of molecular nature and become the dominating states when a significant number of molecules (∼10^6^–10^10^) are required to generate enough Rabi splitting to form polaritons in VSC inside of the Fabry–Perot (FP) cavities [4].

Polariton chemistry has witnessed a notable yet debated discovery concerning the modification of chemical reactions through VSC [2, 5, 6]. However, a comprehensive understanding of how polaritons impact chemistry is still lacking, necessitating a clear mechanistic insight to inform rational design strategies [4, 7]. Using ultrafast spectroscopy, researchers have employed pump-probe and two-dimensional infrared (2D IR) spectroscopy [8–10] to investigate the energy redistribution of polaritons into dark modes, a crucial dynamic process with potential implications for chemical reactions. These works have demonstrated that polaritons can facilitate inter- and intra-molecular vibrational energy transfer [11], and even impede isomerization events [12]. While these findings qualitatively align with the theoretical expectations, achieving quantitative agreements remains a challenge to address.

In our pursuit of advancing the understanding of polariton energy relaxation dynamics, we conducted a detailed investigation into the ultrafast dynamics of MVP formed by the asymmetric *T*_1*u*_ mode of W(CO)_6_ (triply degenerate C–O stretching mode, at 1980 cm^−1^) and an FP cavity. In previous studies [13], we established that LP underwent a transfer to the second excited state of dark modes (*D*_2_), before eventually relaxing to the first excited dark modes (*D*_1_), exhibiting an initial rise followed by decay. This LP-to-*D*_2_ channel was favored due to LP–LP scattering, where the double of LP resonance frequency (2*ω*_LP_) closely matched the 0 to 2 transition of dark modes (*ω*_02_), a mechanism supported by theoretical studies [14].

In this work, we expanded our analysis by incorporating higher excited state transitions into the transfer matrix model (TMM) and employed a kinetic model to fit the dynamics. Our finding revealed that while *D*_2_ was indeed populated, an additional phonon-assisted scattering process occurred, involving the transfer of LP to an IR inactive mode. We proposed that the scatter process from the *T*_1*u*_ mode to the *E*_*g*_ Raman mode, observed in uncoupled W(CO)_6_ system [15–17], was enhanced. Notably, this LP-to-Raman mode scattering process was a third order process, facilitated by solvent phonons. This mechanism is then supported by its dependence on solvents with various carbon chain lengths, which influences the solvent phonon density of states (DOS_ph_). This exciting discovery highlights the involvement of IR-inactive modes in polariton dynamics and shed light to the potential intermediates of the polariton enabled intermolecular vibrational energy transfer [11] and changes of pseudo-rotation dynamics [12].

## 2 Results

**Figure 1:**
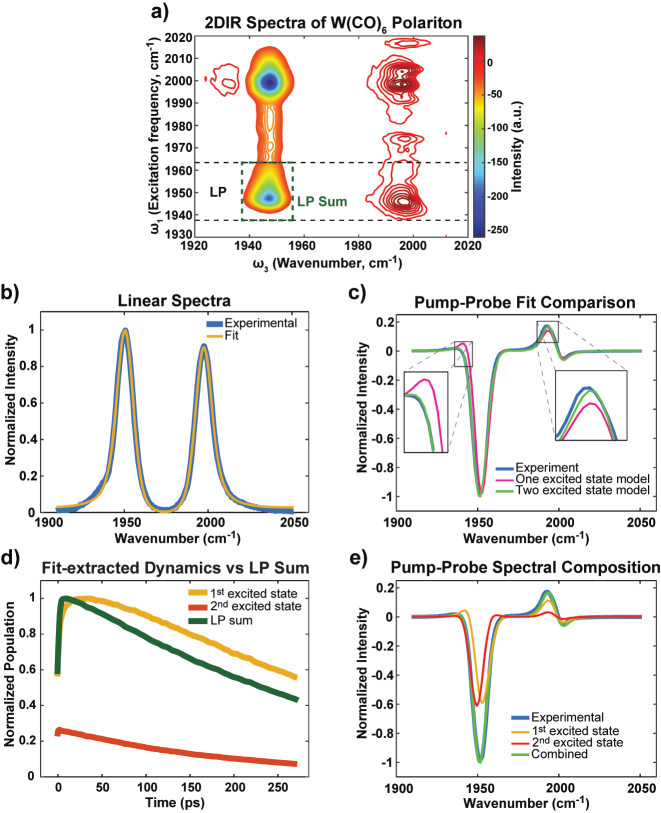
Nonliear spectroscopic signal of polaritons and the representive spectral fitting and extracted dynamics using TMM fitting. (a) Representative broadband pumped 2DIR spectra of W(CO)_6_ polariton system in DCM solvent with Rabi splitting of 55 cm^−1^ at 20 ps time delay. The region demarcated by two dashed black horizontal lines represents the frequency range used for narrowband excitation of LP in the subsequent pump probe experiments. (b) Linear spectra fitted with TMM model (see SI, Spectral Fitting). (c) The narrowband LP pumped pump-probe spectra are fitted with one (*ω*_12_) or two excited state transitions (*ω*_12_ and *ω*_23_) for pump-on component, while other fitting parameters are fixed at the values obtained from the linear spectra fitting in (b). Including the second excited state, *D*_2_ improves the fitting fidelity. The example result is shown here for 15 ps time delay. (d) The relative population dynamics traces of the *T*_1*u*_ excited states compared to LP sum (dashed area in 1a) dynamics trace. LP sum may not faithfully represent the dynamics of the first excited state, *D*_1_. This result emphasizes the need for multi-state TMM model. (e) The corresponding compositions of the pump-probe fit in (c), with *ω*_12_ and *ω*_23_ kept the same as the uncoupled case while fitting the raw transient spectra. They appeared to be close to each other because of their convoluted response when coupled with the cavity.

We conducted a study on the relaxation dynamics of polaritons composed of W(CO)_6_ in dichloromethane (DCM) using 2D IR and tailored pump-probe spectroscopy [18, 19]. The 2D IR spectra revealed four distinct peaks attributed to Rabi splitting contraction and excited state absorption of dark modes (Figure 1a) [8, 20], [21], [22], [23]. Our focus was to understand the dynamics of how LP states relaxed to *D*_1_. To achieve this, we selectively pumped the LP states (indicated by the two dashed lines in Figure 1a) and probed them broadly. The significant absorption peak near *ω*_3_ = 1950 cm^−1^ indicated the population of *D*_1_. By tracking this peak at [*ω*_1_ = *ω*_LP_, *ω*_3_ = 1950 cm^−1^], we could observe the population transfer dynamics from LP to *D*_1_. To extract this dynamic, we employed a semiclassical transfer matrix model (TMM) [8, 20, 24] to fit the linear and pump-probe spectra (Figure 1b and c). The TMM fitting incorporated higher-level transitions of dark modes (such as *ω*_23_) in addition to *ω*_01_ and *ω*_12_ transitions, different from our previous work [20]. This improved model enabled a better agreement between the fitted and experimental dynamics (Figure 1c and inset, S2). By fitting the pump-probe spectra for each time delay, the *D*_1_ and *D*_2_ population dynamics were extracted (Figure 1d).

Similar to our previous observations [13], the rise in dynamics was observed primarily in the *D*_1_ dynamics for narrowband LP excitation but not so for UP (Figures S4 and 1d). While the previous study attributed the early rise to *D*_2_ decaying into *D*_1_, we found that the *D*_2_ dynamics decay much slower than the rise of *D*_1_ (Figure 1d), indicating the involvement of another state in the polariton relaxation process. This observation was further supported by the inability to fit the LP-*D*_1_ dynamics with a kinetic model. Treating the relaxation from the coherent polariton state to the dark state as a fast process, the kinetic model was simplified to only have the ground (*D*_0_), and first (*D*_1_) and second (*D*_2_) excited states (eq. (1)).

(1) $$\frac{\text{d}D_{2}}{\text{d}t}=-k_{21}D_{2};\frac{\text{d}D_{1}}{\text{d}t}=k_{21}D_{2}-k_{10}D_{1};\frac{\text{d}D_{0}}{\text{d}t}=k_{10}D_{1}$$

**Figure 2:**
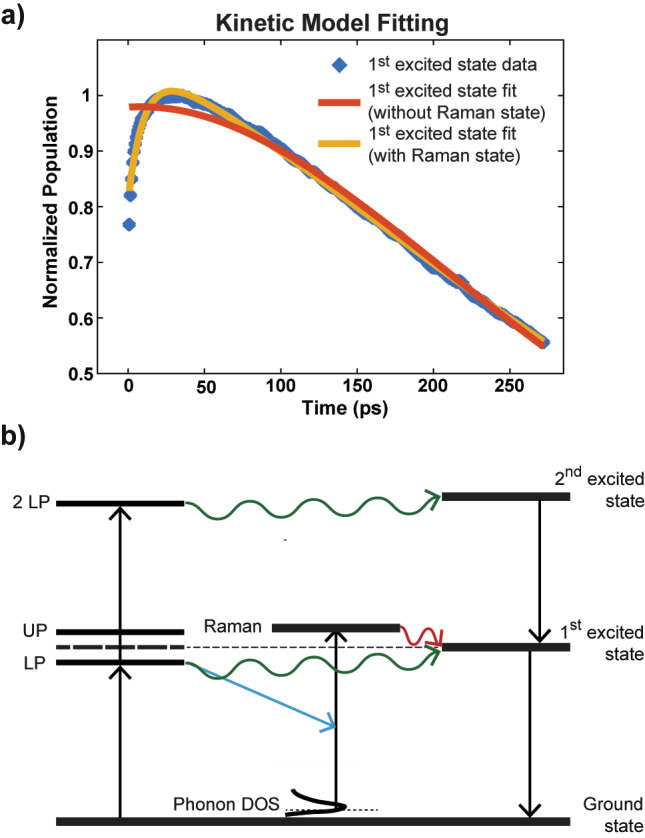
Kinetic fitting results of the dark mode 1st excited state dynamics and the proposed mechanism. (a) The dynamics fitting with and without the Raman pathway. (b) The proposed energy relaxation pathway for the W(CO)_6_ polariton system. The pump excites the LP state which relaxes into the first and second excited state directly (green arrows), or scatters with the solvent phonons to excite the Raman mode (*E*_*g*_) (blue arrow). The *E*_*g*_ mode then relaxes/equilibrates with *D*_1_ (red arrow), following decaying to ground states.

However, this model reached a poor agreement with the LP-*D*_1_ dynamics (orange trace in Figure 2a, Table S2). This is in sharp contrast with the fact that the model fits UP-*D*_1_ and *D*_2_ dynamics well (Figure S4, Table S1). Considering this, the following discussions will primarily focus on the LP-excited *D*_1_ dynamics to understand the reason of this unexpected dynamic.

When we introduced an additional state (*P*_*int*_), feeding into *D*_1_, into the kinetic model (eq. (2))_,_ the dynamics could be adequately described (yellow, Figure 2a).

(2) $$\frac{\text{d}D_{1}}{\text{d}t}=k_{21}D_{2}-k_{10}D_{1}+k_{\text{int}}P_{\text{int}};\frac{\text{d}P_{\text{int}}}{\text{d}t}=-k_{\text{int}}P_{\text{int}}$$

Notably, the lifetime of this additional state (*k*_int_) is ∼11 ps, longer than that of the polaritons (<5 ps) [8], indicating that the new state cannot be a coherent polariton state.

Based on the literature, we hypothesized that this new intermediate state is the IR-inactive Raman *E*_*g*_ mode at 1999 cm^−1^ (Figure S6). Previous experimental results from uncoupled W(CO)_6_ systems have shown that transfer to *E*_*g*_ represents the fastest relaxation pathway for the *T*_1*u*_ vibrational mode, which forms the polaritons here [15, 16]. This process is a third order scattering mechanism that includes solvent phonon modes and occurs within a few ps [16, 17]. Since decaying into the ground state is a slow, multi-order process, the population in the *T*_1*u*_ and *E*_*g*_ modes were observed to equilibrate quickly with each other [16, 17] and thereby exciting either of them should lead to the transfer into the other mode. Extending this argument, we conjecture that due to the initial large *E*_*g*_ population and imbalance between the *E*_*g*_ and *T*_1*u*_ modes following LP relaxation, the *E*_*g*_ Raman mode could transfer population into the *T*_1*u*_ mode at the fast time scale and thereby serve as a relaxation channel for LP, involving an LP-phonon scattering process (Figure 2b).

**Figure 3:**
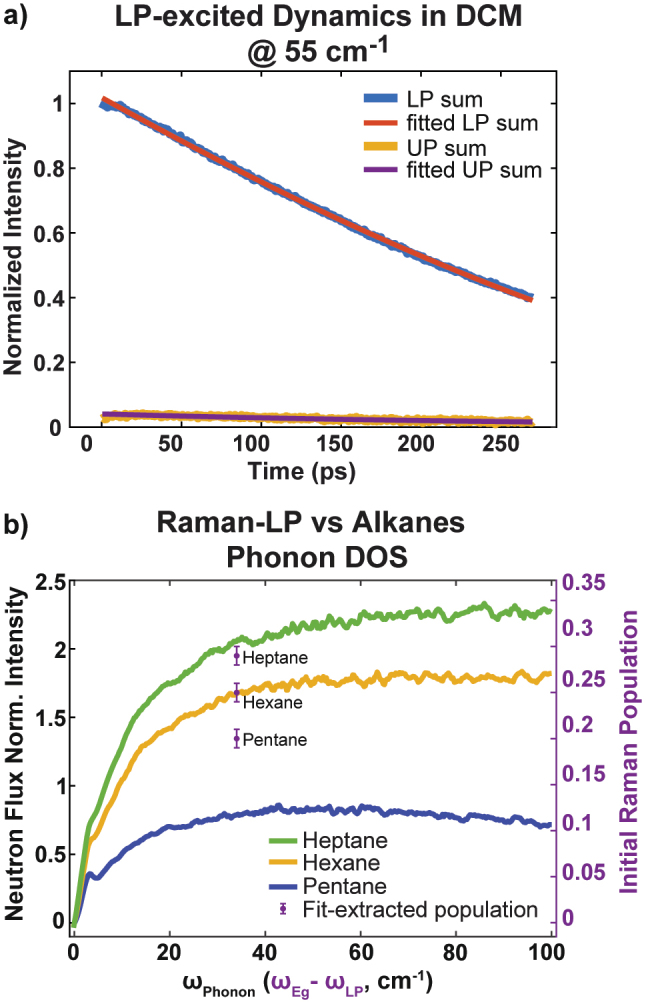
Solvent dependent of the initial Raman state population. (a) Representative fitting results of the combined TMM and kinetic model, where the amplitude of each vibrational states (D_0_, D_1_, D_2_ and *E*_*g*_) are directly determined by the kinetic models (see SI, Combined Spectral and Kinetic Fitting). The fitted and experimental results are compared by integrating the LP-excited pump probe spectra over the LP and UP peak region along *ω*_3_. Initial intermediate (Raman state) population was extracted through this fitting method. (b) The Raman state population at the same Rabi splitting of 34 cm^−1^ increases between polariton systems formed in pentane, hexanes, and heptane solvents, respectively, following their phonon DOS spectra at room temperature.

To directly examine this hypothesis, a time-resolved IR pump Raman probe experiment would be ideal. However, achieving such an experiment is challenging due to the difficulty in designing proper cavity optics that are reflective to IR to form cavities and transmissive to visible light for the Raman measurements. Instead, we focused on examining the relation between the initial population of *E*_*g*_ (*P*_*int*_ (0)) state, obtained from the combined TMM and kinetic model fitting (see SI, example of fitting results see Figure 3a), and the solvent phonon modes involved in the proposed phonon-assisted LP-to-*E*_*g*_ scattering process. This dynamic is a third order process, where LP scatters with one quantum of phonon modes, transferring its energy to the *E*_*g*_ mode. Consequently, the outcome of the scattering, i.e., *E*_*g*_ population, should be linearly proportional to the product of phonon density of states (DOS_ph_) and occupation number (*n*) [25].

To validate this mechanism, we compared the initial population of intermediate states across three alkane solvents, i.e., pentane, hexane, and heptane for the same Rabi splitting (thereby same *ω*_Phonon_ = *ω*_*Eg*_ − *ω*_LP_). These solvents share comparable chemical properties but increasing DOS_ph_ with longer carbon chain (Figure 3b), indicated by the experimental inelastic neutron scattering (INS) spectra. We note that excessively long alkane chains decrease the solubility of W(CO)_6_ and lead to weak coupling regime. Consequently, the options for solvents become constrained under these conditions.

The DOS_ph_ spectra were directly measured from INS measurement at room temperature, with the DOS_ph_ increasing as a function of the number of carbon atoms in the alkane chains. The intermediate state (*E*_*g*_) population of alkanes follows the increase in DOS_ph_, i.e., it elevates as carbon chain length increases (Figure 3b). These experimental findings thus support the claim that the energy transfer from LP to *E*_*g*_ depends on phonon DOS, and thereby is a third order process. This also explains why there is no significant energy transfer from UP to D_1_ (Figure S4): because the energy of *E*_*g*_ and UP modes are near each other, the phonon DOS_ph_ approaches zero for *ω*_ph_ = *ω*_*Eg*_ − *ω*_UP_, inhibiting the energy transfer of UP to *E*_*g*_, an inherent dipole-polarizability interaction with the aid of phonon modes.

In principle, the Raman modes should, theoretically, be affected by other factors including the phonon/LP frequency and temperatures, which can either influence the DOS_ph_ (*ω*_ph_, T) or *n* (*ω*_ph_, T). Our preliminary experimental observations suggest that these anticipated dependencies, primarily of phonon frequency, measured through changes in Rabi splitting, differ from this intuitive expected pattern, with initial Raman population deviating from the DOS_ph_ * *n* spectra (Figure S8, see SI). This deviation implies that certain low frequency modes, contributing to a distinct subpopulation of overall DOS_ph_, play a pivotal role in the phonon-scattering process, as opposed to the inclusive involvement of all phonon modes. Further investigation is warranted to comprehend this aspect.

## 3 Conclusions

This study has opened a promising avenue in vibrational polariton dynamics. While the explicit spectral signature of the *E*_*g*_ modes awaits validation through an IR pump and Raman probe experiment, significant insights have already emerged. Notably, Raman modes have not been considered in polariton dynamics. However, this unexplored Raman pathway illustrates how polaritons could influence chemistry: exciting polaritons can enhance the excited state population of IR-inactive vibrational modes relative to the coupled IR-active mode and thereby promoting the LP-to-Raman energy transfer pathway that is otherwise unfavorable in pure molecular systems. In a case where the reverse energy transfer from Raman mode to IR mode is constrained, the polariton-populated Raman mode could shift chemical dynamics on longer time scales. The involvement of Raman modes holds promise across a broad spectrum, particularly for molecules that have low frequency modes. Whereby, an intramolecular scattering between polariton and the low frequency vibrational modes could occur, thereby impacting the associated molecular dynamics. This mechanism thus could lead to the polariton-enabled energy transfer [11], and pseudo-rotation [12] which remains to be further delved into.

## Supporting Information

Experimental, theoretical, and analytical procedures, additional and detailed results, and discussion of the DOS model (PDF).

## Supplementary Material

Supplementary Material Details

## References

[1] Xiang B., Xiong W. (2021). Molecular vibrational polariton: its dynamics and potentials in novel chemistry and quantum technology. *J. Chem. Phys*..

[2] Garcia-Vidal F. J., Ciuti C., Ebbesen T. W. (2021). Manipulating matter by strong coupling to vacuum fields. *Science (1979)*.

[3] Ribeiro R. F., Martínez-Martínez L. A., Du M., Campos-Gonzalez-Angulo J., Yuen-Zhou J. (2018). Polariton chemistry: controlling molecular dynamics with optical cavities. *Chem. Sci*..

[4] Simpkins B. S., Dunkelberger A. D., Owrutsky J. C. (2021). Mode-specific chemistry through vibrational strong coupling (or *A Wish Come True*). *J. Phys. Chem. C*.

[5] Ebbesen T. W. (2016). Hybrid light–matter states in a molecular and material science perspective. *Acc. Chem. Res*..

[6] Imperatore M. V., Asbury J. B., Giebink N. C. (2021). Reproducibility of cavity-enhanced chemical reaction rates in the vibrational strong coupling regime. *J. Chem. Phys*..

[7] Rider M. S., Barnes W. L. (2021). Something from nothing: linking molecules with virtual light. *Contemp. Phys*..

[8] Xiang B., Ribeiro R. F., Dunkelberger A. D. (2018). Two-dimensional infrared spectroscopy of vibrational polaritons. *Proc. Natl. Acad. Sci. U. S. A.*.

[9] Cohn B., Sufrin S., Chuntonov L. (2022). Ultrafast vibrational excitation transfer on resonant antenna lattices revealed by two-dimensional infrared spectroscopy. *J. Chem. Phys*..

[10] Xiong W. (2023). Molecular vibrational polariton dynamics: what can polaritons do?. *Acc. Chem. Res*..

[11] Xiang B., Ribeiro R. F., Du M. (2020). Intermolecular vibrational energy transfer enabled by microcavity strong light–matter coupling. *Science (1979)*.

[12] Chen T.-T., Du M., Yang Z., Yuen-Zhou J., Xiong W. (2022). Cavity-enabled enhancement of ultrafast intramolecular vibrational redistribution over pseudorotation. *Science (1979)*.

[13] Xiang B., Ribeiro R. F., Chen L. (2019). State-selective polariton to dark state relaxation dynamics. *J. Phys. Chem. A*.

[14] Li T. E., Nitzan A., Subotnik J. E. (2021). Cavity molecular dynamics simulations of vibrational polariton-enhanced molecular nonlinear absorption. *J. Chem. Phys*..

[15] Tokmakoff A., Sauter B., Fayer M. D. (1994). Temperature-dependent vibrational relaxation in polyatomic liquids: picosecond infrared pump–probe experiments. *J. Chem. Phys*..

[16] Tokmakoff A., Sauter B., Kwok A. S., Fayer M. D. (1994). Phonon-induced scattering between vibrations and multiphoton vibrational up-pumping in liquid solution. *Chem. Phys. Lett*..

[17] Arrivo S. M., Dougherty T. P., Grubbs W. T., Heilweil E. J. (1995). Ultrafast infrared spectroscopy of vibrational CO-stretch up-pumping and relaxation dynamics of W(CO)_6_. *Chem. Phys. Lett*..

[18] Grafton A. B., Dunkelberger A. D., Simpkins B. S. (2021). Excited-state vibration-polariton transitions and dynamics in nitroprusside. *Nat. Commun*..

[19] Yang Z., Xiang B., Xiong W. (2020). Controlling quantum pathways in molecular vibrational polaritons. *ACS Photonics*.

[20] Dunkelberger A. D., Spann B. T., Fears K. P., Simpkins B. S., Owrutsky J. C. (2016). Modified relaxation dynamics and coherent energy exchange in coupled vibration-cavity polaritons. *Nat. Commun*..

[21] Ribeiro R. F., Dunkelberger A. D., Xiang B. (2018). Theory for nonlinear spectroscopy of vibrational polaritons. *J. Phys. Chem. Lett*..

[22] Simpkins B. S., Yang Z., Dunkelberger A. D., Vurgaftman I., Owrutsky J. C., Xiong W. (2023). Comment on ‘isolating polaritonic 2D-IR transmission spectra’. *J. Phys. Chem. Lett*..

[23] Duan R., Mastron J. N., Song Y., Kubarych K. J. (2021). Isolating polaritonic 2D-IR transmission spectra. *J. Phys. Chem. Lett*..

[24] Khitrova G., Gibbs H. M., Jahnke F., Kira M., Koch S. W. (1999). Nonlinear optics of normal-mode-coupling semiconductor microcavities. *Rev. Mod. Phys*..

[25] Kenkre V. M., Tokmakoff A., Fayer M. D. (1994). Theory of vibrational relaxation of polyatomic molecules in liquids. *J. Chem. Phys*..

