# Supplementary material for: The role of IR inactive mode in W(CO)6 polariton relaxation process
Source: Nanophotonics. 2023 Nov 8;13(11):2029–34. doi: 10.1515/nanoph-2023-0589 (PMC11501596; doi:10.1515/nanoph-2023-0589)
Supplement: Supplementary file 1 — Supplementary Material Details [file j_nanoph-2023-0589_suppl_001.docx]

The Role of IR Inactive Mode in W(CO)_6_ Polariton Relaxation Process

Oliver Hirschmann^‡,a^, Harsh H. Bhakta^‡,a^, and Wei Xiong*^a,b,c^

a. Department of Chemistry and Biochemistry, University of California San Diego, La Jolla, CA 92093

b. Materials Science and Engineering Program, University of California San Diego, La Jolla, CA 92093

c. Department of Electrical and Computer Engineering, University of California San Diego, La Jolla, CA 92093

**ABSTRACT:** Vibrational polaritons have shown potential in influencing chemical reactions, but the exact mechanism by which they impact vibrational energy redistribution, crucial for rational polariton chemistry design, remains unclear. In this work, we shed light on this aspect by revealing the role of solvent phonon modes in facilitating the energy relaxation process from the polaritons formed of a T_1u_ mode of W(CO)_6_ to an IR inactive E_g_ mode. Ultrafast dynamic measurements indicate that along with the direct relaxation to the dark T_1u_ modes, lower polaritons also transition to an intermediate state, which then subsequently relaxes to the T_1u_ mode. We reason that the intermediate state could correspond to the near-in-energy Raman active E_g_ mode, which is populated through a phonon scattering process. This proposed mechanism finds support in the observed dependence of the IR-inactive state’s population on the factors influencing phonon density of states, e.g., solvents. The significance of the Raman mode’s involvement emphasizes the importance of non-IR active modes in modifying chemical reactions and ultrafast molecular dynamics.

Table of Contents

[Experimental Procedures 3](#_Toc145256246)

[Sample Preparation: 3](#_Toc145256247)

[Pump-probe and 2DIR: 3](#_Toc145256248)

[Classical Polariton Model: 4](#_Toc145256249)

[Fitting Methodology: 5](#_Toc145256250)

[Spectral Fitting 6](#_Toc145256251)

[Kinetic Model 6](#_Toc145256252)

[Combined Spectral and Kinetic Fitting 7](#_Toc145256253)

[Phonon Spectra: 7](#_Toc145256254)

[Additional Results 8](#_Toc145256255)

[Classical TMM fitting: 8](#_Toc145256256)

[Kinetic model and Raman mode: 9](#_Toc145256257)

[Phonon DOS dependence of Initial Raman Population at Different Rabi Splitting: 13](#_Toc145256258)

[Phonon DOS dependence of Initial Raman Population at different DOS: 14](#_Toc145256259)

[Temperature dependence of Initial Raman Population: 15](#_Toc145256260)

[References 17](#_Toc145256261)

Experimental Procedures

Sample Preparation:

The chemicals used in this experiment, i.e., W(CO)_6_, pentane, hexane, heptane, and dichloromethane (DCM), were purchased from Sigma-Aldrich and used without further purification. To prepare the stock solutions, W(CO)_6_ was dissolved in the solvents (pentane, hexane, heptane, and DCM) and then sonicated and kept overnight to reach an equilibrated saturation point. The saturated solutions were then mixed with the additional respective solvents to obtain solutions at various concentrations. Few μLs of the solution were then sandwiched along with a 12-24 μm gradient Teflon spacer between two 96% reflective commercial distributive Bragg reflective (DBR) mirrors (made by Thin Film Corp.) inside a Harrick cell to form a Fabry-Pérot Cavity polariton system. As during the cavity formation step, the volatile solvents evaporated unavoidably from the solutions, the resulting solution had different W(CO)_6_ concentration than the one originally prepared. Thus, Rabi splitting (Ω), which is proportional to the concentration (Ω ∝ $\sqrt{C}$), was used instead as the measurement of the concentration, with maximum Rabi splitting being of the saturated solution. The cell is then placed on a vertically aligned stage, as mentioned in our group’s previous publications,[1] for pump-probe and 2DIR spectroscopy. Time-resolved (t_2_) pump-probe spectroscopic data were collected for both broad and filtered (lower polariton and upper polariton) spectral range of pump pulses and were the primary data analysed in this manuscript.

For lower polariton (LP)/phonon frequency dependence experiments, cavity systems of W(CO)_6_ in DCM and pentane were prepared for multiple Rabi splitting, with minimal detuning, to obtain different LP frequencies. As it was found that the solubility of W(CO)_6_ decreases with the increasing molecular weight of alkanes (resulting in heptane forming the saturated solution with the Rabi splitting of around 34 cm^-1^, while pentane forming that of around 45cm^-1^), for experiments on phonon population dependence at same frequency, concentrations of pentane and hexanes based polariton systems were adjusted to equate to the maximum concentration of W(CO)_6_ in heptane (i.e., Rabi splitting of 34 cm^-1^). To conduct temperature-dependent experiments, a temperature-controlled Harrick cell was used instead of the simple Harrick cell, where an electric and water-based heating/cooling system controlled the cell temperature. Limited by the decrease in solubility of W(CO)_6_ with the decrease in temperature, change of cavity system by thermal expansion of the materials forming the cavity, and the boiling point of the solvent, we used heptane to form polariton systems with equilibrated cell temperature of 22, 32, 42, 52, and 62 °C and three Rabi splittings 22, 27, and 34 cm^-1^.

Throughout the study, the systems used were under strong coupling regime, defined as Rabi splitting (Ω ≥ 20 cm^-1^) being larger than the linewidth of both the cavity (~10 cm^-1^) and the vibrational modes (~5-12 cm^-1^), for all of the solvent systems studied.[2] The angle between pump, probe, and incident normal to the cavity was nearly equal (~7°) to ensure that the states that are excited are same as that are probed. Finally, the polarization between pump and probe beams was kept at magic angle (54.7°) to remove the anisotropy signal from the molecular reorientation in the system.

Pump-probe and 2DIR:

Pump-probe and 2DIR spectroscopic setup and measurement techniques are mentioned in earlier publications by our group.[1], [2] Briefly, the setup uses a Ti:Sapphire solid state laser (Coherent), which produces 4 W at 800 nm with 32 fs pulses at a repetition rate of 1 kHz. The majority of 800 nm pulses (90%) are then converted to 5 μm pulses using an optical parametric amplifier (Light Conversion) and type II AgGaS_2_ difference frequency generation (DFG) crystal. Each 5 μm pulse is then split into probe and pump pulse using a CaF_2_ wedge, resulting in a 1:9 probe to pump intensity distribution. Using an acoustic optical modulator (AOM) based pulse shaper (PhaseTech) each pump pulse is then tailored to the desired frequency range by narrowband spectral shaping and the desired phase for a phase cycling process that removes background scatter. For 2D IR spectroscopy the pump pulse is also split into two pulses with controlled time separation t_1_, using the AOM. A computer-controlled pump delay stage with total range of 325 ps is used to adjust the time delay t_2_ between the pump pulse/s and the probe pulse. The pump pulse beam and the probe beam are then spatially overlapped and focused on the sample using CaF_2_ lenses, the probe beam is magnified using an imaging setup consisting of one CaF_2_ and one ZnS lens (with anti-reflective coating). The beam is then dispersed by a grating to obtain frequency-domain probe spectra (ω_3_) and imaged on a 128x128 HgCdTe (MCT) Focal Plane Array (FPA) detector (PhaseTech).

For 2D IR, the t_1_ time separation between pump pulses is Fourier transformed to obtain ω_1_ axis indicating pump absorption. The pump-probe spectrum, on the other hand, has a single pump pulse exciting the system coherently, with data collected as the difference of probe spectra under pump pulse on and off. The time t_2_ scans the dynamics after excitation by pump, and thus by observing pump-probe and 2D IR spectra evolution along t_2_, the dynamics of the system is determined. In this study, primarily pump-probe spectra with spectral filtered pump pulses, i.e., AOM-filtered pump pulses were used. To do so, first full spectral range 2D IR were obtained in order to determine the frequency range for UP and LP on the ω_1_ axis. That range was then applied to the AOM to generate tailored-pump pulses for pump-probe spectroscopy.[3]

Classical Polariton Model:

A similar semiclassical approach was used to describe polariton systems as in the previous publications by our group[2] as well as by Dunkelberger et al.[4] In that, this approach involved quantized molecular vibrations in classical electromagnetic field. If the molecular system is treated as comprised of uncoupled quantum harmonic oscillators, then its linear susceptibility is given as:[5]

$$\chi\left( \omega\right)= k \Sigma_{ab}P\left( a \right)\frac{\left| \mu_{\mathrm{ab}} \right|^{2}\omega_{ba}}{\omega_{ba}^{2}-\omega^{2}-2i \Gamma_{ab}\omega}$$

Where k is the proportionality constant which depends on the density of molecules, P(*a*) is the population in state *a*, ω*_ba_* is the frequency for transition between states *b* and *a*, Γ*_ab_* is its dephasing rate, and μ*_ab_* is its transition dipole moment. For the harmonic oscillator, it is known that the relation among the transition dipole moments is $\mu_{i-1,i}=\sqrt{i} \mu_{01}$. However, as W(CO)_6_ is an anharmonic system, and in certain solvent systems, we might expect that this relation is not rigorously followed. In fact, if this relation was considered in fitting the W(CO)_6_ polariton data, it could not adequately describe the system (Figure S1, $\mu_{12}^{2}=2\mu_{01}^{2}, \mu_{23}^{2}=3\mu_{01}^{2}$). From Figure S1, the other transition dipole relation such as ($\mu_{12}^{2}=1.414\mu_{01}^{2}, \mu_{23}^{2}=1.732\mu_{01}^{2}$) could not describe it either. When $\mu_{12}^{2}=1.189\mu_{01}^{2}, \mu_{23}^{2}=1.316\mu_{01}^{2}$ or $\mu_{23}^{2}=\mu_{12}^{2}=\mu_{01}^{2}$, both the pump-probe spectra and the time dynamics can be reasonably described with this semiclassical approach (Figure S1a and c). Considering that the time dynamics is similar among them (Figure S1b) and that phenomenologically nearly equal dipole relations can describe the polariton system better, we treated molecular susceptibility without explicitly considering the different transition dipole moment of different excitations, with $A_{i}=k/\varepsilon_{0} P\left( i \right)\left| \mu_{i,i+1} \right|^{2}\omega_{i+1,i}$ comprising this relation.

$$\chi\left( \omega\right)=\Sigma_{i}\frac{\varepsilon_{0} A_{i}}{\omega_{i+1,i}^{2}-\omega^{2}-2i \Gamma_{i,i+1}}$$

The susceptibility χ(ω) is then used to calculate the frequency dependent complex dielectric function ε(ω) of the solution, with the solvent dielectric constant being used as background ε_∞_. The refractive index *n* and the absorptivity coefficient α are calculated from the dielectric function and used to describe the solution inside the cavity formed by two ideal mirrors, with reflectivity *r* (transmission t) and cavity length L (in cm). The transmission T of the classical light, at normal incidence angle θ, through the cavity is then analytically solved using a transfer matrix model (TMM).

$$\varepsilon\left( \omega\right)=\varepsilon_{\infty}+\frac{\chi\left( \omega\right)}{\varepsilon_{0}}=\varepsilon_{\infty}+\Sigma_{i}\frac{A_{i}}{\omega_{i+1,i}^{2}-\omega^{2}-2i \Gamma_{i,i+1}\omega}$$

$$n\left( \omega\right)=Re\sqrt{\varepsilon\left( \omega\right)}, \alpha\left( \omega\right)=4\pi\omega\mathrm{Im}\sqrt{\varepsilon\left( \omega\right)}$$

$$T\left( \omega\right)=\frac{t^{2} e^{-\frac{\alpha(\omega)L}{\cos\theta}}}{\left| 1-r e^{-\frac{\alpha\left( \omega\right)L}{\cos\theta} +(4\pi\omega n\left( \omega\right)L\cos\theta+\phi) i} \right|^{2}}$$

$$I\left( \omega\right)\propto\int_{-\infty}^{\infty} T\left( \omega^{'} \right) e^{\frac{-\left( \omega-\omega^{'} \right)^{2}}{2\sigma^{2}}} d\omega'$$

Although there is a slight angle between the plane of the two mirrors, as was described the ϕ parameter in previous publications, we found it not to be required and it was kept at ϕ=0.[2] Finally, the observed intensity spectrum *I(ω)* is calculated using the gaussian-convoluted transmission spectra to account for the convolution of the probe beam profile, which is gaussian, with the transmitted signal, in the observed heterodyned signal, and the experimental imaging setup. To improve the speed of fitting the analytical intensity equation to experimental data, the limits of convolution were restricted to ± 3σ, i.e., 99.9% confidence interval, and numerical integration was used. Overall, in comparison to earlier approaches in modelling of transmission spectra, the angle parameter and the experimental σ parameter, which indicates the unsharpness in the measured spectra due to magnification and the pixelated detector, were also included.

***Figure S1.*** The transition dipole moment relation in susceptibility for harmonic oscillator is tested by changing the ratio among the transition dipole moments and fitting the resulting model on the transient spectra for coupled W(CO)_6_ dichloromethane (DCM) solvent at Rabi splitting of 55 cm^-1^ (same as in Figure 1 and Figure S4). For harmonic oscillator, $\mu_{12}^{2}=2\mu_{01}^{2}, \mu_{23}^{2}=3\mu_{01}^{2}\left( 2,3 \right)$is theoretically expected, while $\mu_{12}^{2}=1.414\mu_{01}^{2}, \mu_{23}^{2}=1.732\mu_{01}^{2}$ (1.4,1.7) and $\mu_{12}^{2}=1.189\mu_{01}^{2}, \mu_{23}^{2}=1.316\mu_{01}^{2}$(1.2,1.3) are further square roots of the original relation, and $\mu_{23}^{2}=\mu_{12}^{2}=\mu_{01}^{2}$ (1,1) describe a scenario where it does not play a significant role in the susceptibility. As seen in a), the transient pump-probe spectra are not fitted accurately by (2,3) and (1.4,1.7) relation, while well enough by (1.2,1.3) and (1,1), indicating that higher dipole ratios could not adequately describe the system. In b) the extracted population P(i) are plotted for the 1^st^ and 2^nd^ excited states for these relations over time after excitation, and c) shows the goodness of the fits at each time, validating whether the extracted data are reasonably describing the dynamics of the system. As seen in b), the dynamics of the (1,1), (1.2,1.3), and (2,3) are similar, while that of (1.4,1.7) has an unusual 2^nd^ excited state decay, which matches with the decrease in its R^2^ value at lower time. But regardless, all of them have the increase in early 10 ps, corresponding to the Raman excited states contribution to the 1^st^ excited state population.

Fitting Methodology:

Spectral Fitting

In this study, the analytical transmission equation was fitted with the experimental transmission spectra to recover the amplitudes A_i_ for various vibrational excitations ω_i+1,i_, and thus the population P(i) of the individual states. To do so, all degrees of freedom in the system had to be considered. There are 3 parameters (A_i_, ω_i+1,i_, and Γ_i,i+1_) for each vibrational state. In addition, there is the background ε_∞_, which is defined as the dielectric constant of the solvent, the reflectivity of the mirrors *r* (if the two mirrors have different reflectivity, r must be replaced with $\sqrt{r_{1}r_{2}}$, the same applies to the transmission), the angle of incidence θ, the phase shift ϕ due to non-parallel mirroring, σ for the experimental conditions, the cavity length L, and initial Intensity I_o_. In total, a doubly excited system, for example, has a total of 16 parameters. Of them, ω_i+1,i_, θ, r, and ε_∞_ are known precisely and are therefore constant, while L, Γ_i,i+1_ and σ are known less precisely and are therefore restricted to certain values. Although ϕ is not known, there was no need to use it during the fitting and so 0 was kept. A_i_, on the other hand, was the primary parameter to be extracted, so there are 9 parameters that can be used to fit the data. Here the NonlinearModelFit (NMF) function in Mathematica was used with the fitting method QuasiNewton, to fit the analytic intensity function with the experimental data.[6]

To simplify the problem and get accurate results, a three-step fitting methodology was implemented. First, the linear pump off spectrum was fitted with one ground-state Lorentzian oscillator and the remaining experimental parameters, so that the 5 parameters A_0_, Γ_01_, σ, L, and I_o_ were determined. Second, the pump-probe spectra were fitted with the intensity equation of the ground state Lorentzian oscillator (pump off) subtracted from the intensity of the multi-state Lorentzian oscillator (pump on). Since the 5 parameters (A_0_, Γ_01_, σ, L, and I_o_) are already known from the first fitting, they are kept constant here and the A_i_ and Γ_i,i+1_ parameters of the excited states are fitted. Here, the population conservation condition is imposed as Σ_i=0_ A_i_=A_0_ i.e., the ground state population in the pump-on equation is A’_0_= A_0_ - Σ_i=1_ A_i_ (For the dipole moment related data in Figure S1, the dipole moment relation is considered when conserving the population, Σ_i=0_ P(i)=P_o_ and A_i_∝P(i) μ_i,i+1_). A two-parameter search approach was implemented for both steps mentioned here: First, good initial parameters had to be found manually, which were then fitted using NMF fitting. The other search approach involved randomly generated multiple (on the order of hundreds) reasonable initial parameters. These were then fitted individually and only the best fits (R^2^>R_tol_ (0.97)) and matches were then subsequently used. Third, the time evolution of pump-probe spectra was adjusted by fitting the pump-probe spectrum at each time step measured, similar to the approach used by Dunkelberger et al. in their recent publications.[4], [7] As the Γ_i,i+1_s were already obtained, only the excited states A_i_ were fitted here. During all these three steps, the quality of the fit was both viewed manually and through the R^2^ value, to ensure that the fit is physically valid. For linear spectra, fitting quality of the peak heights of the two polariton peaks were prioritized rather than its fullwidth-half-max. For pump-probe spectra, the accuracy of all main peaks was prioritized while at the same time ensuring that no additional non-experimental peak appeared. Finally, for the time evolution, it was checked whether the fitted spectra followed the trend of the experimental data. Here, the R^2^ fit over time was also considered to ensure the validity of the fitted parameters. Only after all these conditions were met, the fitted parameters were used further. Given the high number of degrees of freedom, multiple parameter sets can fit the data equally well. For example, multiple resonator lengths (L) denoting different order of resonator modes can describe the data equally well and are thus possible physical scenarios to the same extent. Fortunately, it is observed that all good sets of fitted parameters have exactly similar time dynamics of A_i_, only their value scale differs. Thus, for the further analysis of the extracted data, only the normalized data is used.

Kinetic Model

The extracted excited state population data is then fitted with the multi-state kinetic model. In this study, a 4-state vibrational relaxation model was used, which includes one ground state, two vibrational excited states, and one Raman excited state. All the vibrational excited states decay into the subsequent lower vibrational state until ground state and the first Raman excited state decays into the first vibrational excited state.

$$\frac{dP_{2}}{dt}=-k_{21}P_{2}, \frac{dP_{r_{1}}}{dt}=-k_{r_{1}1}P_{r_{1}}$$

$$\frac{dP_{1}}{dt}=k_{21}P_{2}-k_{10}P_{1}+k_{r_{1}1}P_{r_{1}}$$

$$\frac{dP_{0}}{dt}=k_{10}P_{1}$$

Since the extracted ground state population (A’_0_= A_0_ - Σ_i=1_ A_i_) does not include the Raman state contribution and otherwise is redundant information from the population conservation condition, only the extracted first and second excited states were fitted. As the extracted excited state data is normalized, an additional proportionality constant was added to the analytically solved P_i_(t) to fit them. The explicitly solved form of the analytical equations were then used to fit the excited state A_i_s and to obtain the rate constants and initial populations of the excited states. We used the relaxation rates found for excited states of the uncoupled system as an initial guess to describe the coupled W(CO)_6_, and obtained similar fitted relaxation rates for the excited states ($k_{r_{1}},k_{10},k_{21}$). Based on harmonic oscillator approximation, the relaxation rates should be k_21_= 2 k_10_ and k_32_= 3 k_10_. However, we found that k_21_ ~1.4 k_10_ and k_32_ ~2 k_10_ can best describe the relaxation rate of both the coupled and uncoupled W(CO)_6_ (Table S2 for W(CO)_6_ in dichloromethane). This deviation is possibly due to the anharmonicity of W(CO)_6_, the non-linearity of the relaxation pathways for W(CO)_6_, which, among other solvent-dependent nonlinear processes, involves the coupled relaxation of the T_1u_ mode with the E_g_ Raman mode.[8] From the fitting, we also obtained the initial Raman ($P_{r_{1}}(0)$), first ($P_{1}(0)$) and second ($P_{2}(0)$) excited state population. Considering that the population of Raman excited state was not involved in the original conservation condition of populations (Σ_i=0_ A_i_=constant) but is instead extracted from the relaxation rate of the extracted first excited state population trace, the extracted population traces and thereby the initial excited populations might not be completely representative. One possible approach to remediated this would have been to include an additional Raman state in the population conservation (A­_r1_ + Σ_i=0_ A_i_=constant) for the ground state population. However, doing so resulted in unreproducible and unphysical traces. This is likely since the Raman state, by means of the early rise part in the first excited state trace, is only relevant for early time, while adding it for all time in the conservation condition, introduces an extra degree of freedom which is not needed.

Combined Spectral and Kinetic Fitting

To account for the Raman population, the spectral and kinetic methodologies were combined and two dimensional fitting, i.e. along frequency and time, of the transient p opulation dynamics was performed. Specifically, the analytical form of the populations (P_i_(t)), obtained from solving the kinetic model, was substituted in the TMM model to describe the amplitude (A_i_ ∝ P_i_(t)), and thereby formulating a time and frequency dependent dielectric function *ε(ω,t)*, transmittance *T(ω,t)*, and pump-probe signal *I_pumpon_(ω,t) - I_pumpoff_(ω,t).*

$$\varepsilon\left( \omega,t \right)=\varepsilon_{\infty}+\Sigma_{i=0}^{n=2}\frac{{c_{i}P}_{i}(t)}{\omega_{i+1,i}^{2}-\omega^{2}-2i \Gamma_{i,i+1}\omega}$$

Here, c_i_ is the proportionality constant between the amplitude A_i_ and the normalized population P_i_(t). Similar to the direct spectral fitting method, the overall population, including Raman state, is conserved (P_r1_(t)*c_0_ + Σ_i=0_ P_i_(t)*c_i_ = constant = P_0_(t=0) *c_0_). To avoid fitting large number of spectral and kinetic parameters but obtain a reasonable fit, we followed the methodology in the previous two subsections, i.e., fitted one linear and the pump-probe spectra at the late time to obtain the required spectral parameters, which (except for Ai(t>0)) were fixed for the combined fitting, and fitted the extracted amplitude trace from transient pump-probe fittings with kinetic model to obtain the kinetic parameters, which were used as an initial guess for the combined fitting. We found that including the Raman population in the conservation condition caused little change in the kinetic parameters in comparison with the kinetic parameters obtained without involving Raman in the conservation condition but extracting it from the kinetic trace of the first excited state. Regardless, involving Raman in the conservation condition ensures the physicality of the extracted values as overall population must be conserved. Thus, with this fitting model, we optimized the pump-probe fitting at each timestep all at the same time and obtained the initial populations for all the excited states ($P_{r_{1}}\left( 0 \right), P_{1}\left( 0 \right),P_{2}(0)$), which were then normalized with total excited states initial population ($P_{r_{1}}\left( 0 \right)+P_{1}\left( 0 \right)+P_{2}(0)$) for their analysis.

Phonon Spectra:

The inelastic neutron scattering (INS) spectra for pentane, hexane, heptane, and dichloromethane were collected by Luke Daemen at Oak Ridge National Laboratory. Consistent with the measured polariton data, the INS spectra were measured at room temperature, 22°C. Since our goal was to compare phonon spectra of alkanes relative to each other, the INS spectra were collected for same number of moles of alkanes and were normalized with neutron flux, which was kept constant among all measurements. The collected neutron scattering spectra were then Bose factor corrected to obtain the phonon density of states (DOS_ph_) spectra (Fig 3b). Considering that the third order scattering process is proposed to be dependent on the phonon population, the phonon population spectra were obtained by multiplying the DOS_ph_ spectrum with a phonon occupation number n, given as Boltzmann distribution at room temperature. The reason for using Boltzmann distribution is that Bose-Einstein (BE) distribution gives a non-zero population for ω=0, which essentially indicates the occupation of state with zero density, and thereby being unphysical. However, previous literatures have claimed to reproduce the experimental specific heat spectra of liquids using BE distribution as occupation number.[11]–[13] Yet, the assumption of using solid-phonon BE distribution for liquids is debated, with the opposing argument being that the liquids are classically described instantaneous normal modes (INMs) and thereby should not be treated like solid-phonons[14]. As such, here we describe the occupation number with Boltzmann distribution instead of BE distribution.

Additional Results

Classical TMM fitting:

***Figure S2.*** Adjusted R^2^ values over time for a one excited state model and two excited state models fitting of W(CO)_6_ polariton dynamics in dichloromethane (DCM) solvent. The one excited state model cannot fit spectra at all times equally and thus inadequately captures the polariton dynamics, while the two excited state model is consistent in its fitting and thus in capturing the dynamics of the polaritons.

The collected spectra were fitted with a classical transfer matrix model. The model treats the solution of W(CO)_6_ as a media composed of background solvent and a homogeneously distributed Lorentzian oscillator for each vibrational transition. Maxwell’s equation is then analytically solved for this media inside the cavity formed by two ideal mirrors (equations given in the method section). Dunkelberger et al. used a similar model for the analysis of sodium nitroprusside (SNP) and W(CO)_6_.[7] For simplicity, their study was conducted such that only first excited states can be excited and thereby can be described with two Lorentzian oscillators (for ω_01_ and ω_12_ transitions). The one parameter required for fitting is the ratio between the first and ground state population. In our study, however, using a two-oscillator model cannot adequately fit the pump-probe spectra (Figure 1b, for dichloromethane) and also have the variation in the R^2^ values of the transient spectra fitting over time (Figure S2). Both of which indicate that the dynamics of the polaritons could not be described using the first excited state only. When an additional oscillator for transition ω_23_ was introduced, pump-probe spectra fitted well (Figure 1b) and the of R^2^ values of the transient spectra fitting over time was also consistent, indicating that the two excited state model can accurately describe the polariton dynamics (Figure S2). Adding additional oscillators is not surprising if we consider that W(CO)_6_ has a large dipole moment, higher excited states can be observed to be excited outside cavity, and that the EM field is large inside the cavity to facilitate multi-level excitations.

Here we note that, in the scenario that the 1^st^ excited state is the only state excited, as is the case for the composite W(^12^CO)_6_ and W(^13^CO)_6_ cavity system, the traditional way of using LP sum and MP sum can indeed represent the dynamics of the 1^st^ excited states of W(^12^CO)_6_ and W(^13^CO)_6_ system, respectively (Figure S3).[15]

*****Figure S3.*** Classical Transfer Matrix model fitting of W(^12^CO)_6_ and W(^13^CO)_6_ polariton system in a 1:1.2 dichloromethane (DCM) and hexane solution. a) linear spectrum data at time (t_2_=238.25 ps) was fitted with two-state (ω_01_s) model. b) the pump-probe spectrum at same time was fitted with four-state (ω_01_s and ω_12_s) model, while constraining the repeated parameters from the linear spectrum fitting. The parameters from linear and pump-probe spectral fitting were then used as initial guess to fit the population change with time: c) shows the adjusted R^2^ value (goodness of the fitting) for each transient spectrum fit with time, indicating the confidence in the fitted transient spectra; and d) shows the comparison of the extracted 1^st^ excited states dynamics (W(^12^CO)_6_ and W(^13^CO)_6_) with the usual method of using MP and LP sums. Here, MP and UP sums are similar to the extracted W(^12^CO)_6_ 1^st^ excited state dynamics, while LP sum is that to that of W(^13^CO)_6_.

Kinetic model and Raman mode:

The dynamics of the 1^st^ excited state, when the system is excited by LP and full spectral range pumps, is different from that of the uncoupled system (Figure S4 for DCM). Although the 2^nd^ excited state had similar dynamics, the 1^st^ excited state had an unusual early rise in the population for LP and broadband excitations (Figure S4). When fitted with a simple decay kinetic model, where each excited state decays into the consecutive lower energy state, this early rise could not be explained by the model (Figure S5), implying that it involves more than that due to the decay of 2^nd^ excited state into the 1^st^ one. However, the LP-excited W(CO)_6_ polariton system after the first 30 ps (Figure S5) can be fitted well by this model and had moderately similar lifetimes as the uncoupled cavity system (Table S2), indicating that the late time dynamics could be explained by the 2^nd^ excited state decaying into the 1^st^ excited state. To explain the unusual early rise, we here propose the phonon-induced excitation of a near-in-energy Raman mode and its decay into the 1^st^ excited state (Figure 2). This is reasonable considering that previous studies on an uncoupled W(CO)_6_ system found that an IR-inactive Raman-active E_g_ mode (Figure S6) plays a key role in the dynamics of the IR-active T_1u_ mode,[16] which is the mode coupled to the cavity here. To incorporate the Raman mode, the kinetic model involving simple relaxation dynamics was modified to involve an external relaxation from the Raman mode into the first excited state (see Kinetic Model Subsection in Experimental Procedures). Using this, comparison of excited state population lifetimes with that of the uncoupled system indicated that other than the involvement of Raman mechanism at early times, the coupled system had similar behavior as the uncoupled system (Figure S7, Table S2).

***Figure S4.*** The dynamics of the extracted excited states of the coupled W(CO)_6_ polariton systems in dichloromethane (at 55cm^-1^ Rabi splitting) when excited by broadband (full-spectral), LP (narrowband pump), and UP pump pulses. The UP excitation is similar to the uncoupled system (Figure S6). On the other hand, the broadband and LP excitations has the initial rise which is not observed in uncoupled system.

*****Table S1.*** Kinetic-fit extracted initial excited states population of the coupled W(CO)_6_ polariton system in dichloromethane (at 55cm^-1^ Rabi splitting) for broadband excitation, LP and UP excitation (narrowband pump). Both broadband and LP excitation show an energy transfer into the intermediate (Raman) state, while UP pump does not show this transfer. The corresponding dynamics are shown in figure S4.

*****Figure S5.*** LP-excited dynamics of the DCM-based W(CO)_6_ polariton system (Rabi Splitting of 55 cm^-1^) is fitted using the regular kinetic model involving the decays within the T_1u_ vibrational mode. The kinetic model fitting for all time (>0 ps, yellow & blue) cannot fit the early rise of the 1^st^ excited state adequately and thus the involvement of the E_g_ Raman mode is essential for fitting the earlier time dynamics (Figure 2). On the other hand, the regular kinetic model can adequately describe the late time dynamics (for time >30 ps, red & violet).

***Table S2.*** Kinetic fit parameters for coupled and uncoupled W(CO)_6_ in dichloromethane (55 cm^-1^ Rabi Splitting). The excited states and Raman state are the initial population obtained from the kinetic model. Compared to the uncoupled system (Outside cavity), the coupled system with Raman-mode involved combined spectral and kinetic fitting (Inside cavity) showed very similar lifetimes (𝜏_10_, and 𝜏_21_), albeit with more initial population in higher excited states. The coupled system without Raman-mode kinetic fitting for time >30 ps (Inside cavity Fig S5, for >30ps) had similar but slightly lower lifetime compared to both the Inside and Outside cavity. While, the one for time >0 ps (Inside cavity Fig S5, for >0ps) does not fit the data well enough and thus is expected to have very different fitting parameters.

***Figure S6.*** Normalized Raman spectra for W(CO)_6_ in DCM, pentane, hexanes, and heptane. The Raman active E_g_ mode for all solvents is at ~2000cm^-1^, which in previous studies[16] of uncoupled W(CO)6 was demonstrated to be involved in the relaxation dynamics of Raman-inactive vibrational T_1u_ mode (~1976-1985 cm^-1^). The Raman active A_1g_ mode around ~2100 cm^-1^ was not observed to play role for the uncoupled system and as such was not considered in here.[16]

***Figure S7.*** a) The transient spectrum (pump-probe spectrum) of the saturated W(CO)_6_ in dichloromethane. b) The corresponding fitted kinetics for the first and second excited state (ground state bleaching was also fitted to ensure the accuracy but not plotted to avoid confusion).

***Table S3.*** Kinetic fit parameters for uncoupled W(CO)_6_ in pentane, hexane, heptane, and dichloromethane, showing similar 𝜏_1_ as extracted from the cavity-coupled case.

Phonon DOS dependence of Initial Raman Population at Different Rabi Splitting:

The modified kinetic model was used to extract the initial excited state populations of the two vibrational excited states and the first Raman excited state. Considering that the ground state population is redundant information (see method) calculated from the excited state populations using population conservation condition, and that the fittings of the excited states have some error in the absolute values of the populations calculated, we did not use the ground state population for analysis. Instead, the initial excited state populations were normalized with respect to the total excited state population to compare them at different Rabi splitting. Here, we note that the pump intensity was kept same for each congregate of data compared (Figure S8a, for example) by running all the corresponding experiments on the same day and conditions. As such, all comparative data were collected under the same pump fluence and thereby allowing a reasonable comparison using the relative initial excited state populations.

Different Rabi splitting data were collected and analysed for LP excitation of W(CO)_6_ polaritons formed in DCM and pentane solvents (Figure S8). Since the two solvent systems have different physicochemical properties, studying the Raman mode dependence on their Rabi splitting would validate our hypothesis of the LP dependent scattering process. From the fitted kinetic parameters (Table S4), the relative second excited state initial population seems to follow opposite trends for pentane and DCM. Although there is no clear reason for this opposing trend, we suspect that the larger linewidth and different anharmonicity of the W(CO)_6_ in DCM plays a role in allowing for higher order excitations even at large Rabi splitting, and the trend in pentane is likely due to lack of excitation of the lower order transitions at larger Rabi splitting as it has narrower linewidth. Unlike the relative second excited state initial population, the relative initial population of the first excited state decreases for DCM and is similar for pentane with increase in Rabi splitting. This is due to the combination of their population trends in second excited states and the increasing trends in Raman mode excitation. The lifetimes for the first excited state (𝜏_1_) relaxations are similar for both pentane and DCM at different Rabi splitting. As seen in Tables S2-4, the values of the relaxation lifetimes are also similar for coupled and uncoupled cavity systems.

***Table S4.*** Kinetic fit parameters for cavity-coupled W(CO)_6_ in dichloromethane and pentane, shown for different Rabi splitting.

Even though the trends of vibrational excited state populations are different, both solvent systems have an increase in Raman population with the increase in Rabi splitting. When compared to their respective phonon population spectra (Figure S8a-b), this clear increase cannot be substantiated adequately, i.e., the increase in the Raman population does not follow the decrease in the population spectra. Considering that the INS spectra involve all phonon degrees of freedom, it is possible that the mechanism of LP-scattering proposed here would involve more of a contribution from one of these factors but not others. However, to resolve them, quantum mechanical (QM) simulation of the system would be needed. The other possibility is that the current analysis of the INS spectra did not correct for the Debye-Waller factor, which accounts for the different neutron scattering response of different atoms, which for hydrogens in alkanes is quite large, and thereby significantly affecting the phonon DOS spectra [17]. Thus, we expect to have a significant Debye-Waller factor contribution in our INS spectra, which has not been corrected, and would need computational or experimental resources to address[18]. With the limited number of resources currently available, this is left for future investigations.

***Figure S8.*** Comparison of phonon population spectra (phonon density of states * occupation number) with the initial Raman population at various Rabi splitting for a) DCM and b) pentane-based W(CO)_6_ polariton systems.

Phonon DOS dependence of Initial Raman Population at different DOS:

To investigate the influence of the phonon density of states, the LP excitation of polariton in different solvents were compared while keeping the Rabi splitting constant. The series of solvents that are particularly suitable for this purpose are alkanes, as not only are their physiochemical properties essentially comparable, but also their IR vibrational peaks are similar, making the only possible trend observed among them, when at same concentration and under similar experimental conditions, directly attributable to their different phonon spectra. As the phonon spectra increases with the longer alkanes (Figure 3b), the Raman population is expected to increase from pentane to heptane. The relative initial Raman excited state populations, indeed, qualitatively followed the phonon intensity trend for pentane, hexane, and heptane solvent-based W(CO)_6_ polariton systems (Figure 3b, Figure S9, Table S5). This indicates that the phonon intensity does determine the excited population of the Raman mode rather than anything else such as the solvent’s IR spectrum. Other than the Raman state population, the relative first excited state population decreases from pentane to heptane, while that of second excited state is slightly increasing from pentane to heptane. Here, we note that the first and second excited state population distribution can vary depending on the pump beam intensity and the focus condition, as observed in pentane data of Figures 3b and S8b, shown in Tables S4 and S5. Thus, for all data being compared in the same figures or tables, they were collected at the same day under the exact same conditions, to avoid complications caused by day-to-day laser drifts. The excited state population relaxation lifetimes (τ_1_) are similar to the uncoupled cavity data (Table S2), while the Raman state population lifetimes are similar for all solvents (Tables S4 and S5), indicating that the coupled system has similar behavior as uncoupled systems, and that the Raman mode lifetime is possibly solvent and Rabi-splitting independent. In conclusion, phonons and Raman mode play a decisive role in the polariton dynamics of W(CO)_6_ and must be considered in future explorations of polaritonic dynamics.

***Table S5.*** Kinetic fit parameters for cavity-coupled W(CO)_6_ in alkanes (pentane, hexanes, and heptane) at 34cm^-1^ Rabi splitting.

Temperature dependence of Initial Raman Population:

Considering that phonon DOS are sensitive to changes in temperature, the initial Raman population is hypothesized to be temperature dependent and with similar dependence as the phonon DOS. For temperature dependent measurements, W(CO)_6_ based polariton systems were prepared in heptane solvent, as it had a higher boiling point in comparison to DCM, pentane, and hexane. Pump-probe data were collected at five temperatures (295:10:335 K) and three Rabi splittings (22, 27, and 34 cm^-1^) and analysed to obtain the initial Raman population (Fig S9). As seen in Fig S9, the initial Raman population decreases with the increase in temperature for all three Rabi splitting. Also, as the ω_Eg_-ω_LP_ (=ω_Phonon_) increases, the initial Raman population also increases, agreeing with the trend for pentane and DCM based W(CO)_6_ polariton systems (Fig S8). Additionally, with the increase in ω_Eg_-ω_LP_, the slope of the initial Raman population vs temperature decreases. Currently, we are lacking the INS spectra at different temperatures and do not have any means to obtain them, and as such we would have to be content with the temperature dependent trend of the initial Raman population as a possible indication of the phonon dependence.

***Figure S9.*** Temperature dependence of the initial Raman population for cavity-coupled W(CO)_6_ in heptane at 34, 27 and 21cm^-1^ Rabi splitting. The decreasing trend with increase in temperature or Rabi splitting is observed.

***Table S6.*** Temperature dependent kinetic fit parameters for cavity-coupled W(CO)_6_ in heptane at 34, 27 and 21cm^-1^ Rabi splitting.

References

[1] B. Xiang, Z. Yang, Y. You, and W. Xiong, “Ultrafast Coherence Delocalization in Real Space Simulated by Polaritons,” *Adv Opt Mater*, vol. 10, no. 5, p. 2102237, Mar. 2022, doi: 10.1002/adom.202102237.

[2] B. Xiang *et al.*, “Two-dimensional infrared spectroscopy of vibrational polaritons,” *Proceedings of the National Academy of Sciences*, vol. 115, no. 19, pp. 4845–4850, May 2018, doi: 10.1073/pnas.1722063115.

[3] Z. Yang, B. Xiang, and W. Xiong, “Controlling Quantum Pathways in Molecular Vibrational Polaritons,” *ACS Photonics*, vol. 7, no. 4, pp. 919–924, Apr. 2020, doi: 10.1021/acsphotonics.0c00148.

[4] A. D. Dunkelberger, R. B. Davidson, W. Ahn, B. S. Simpkins, and J. C. Owrutsky, “Ultrafast Transmission Modulation and Recovery via Vibrational Strong Coupling,” *J Phys Chem A*, vol. 122, no. 4, pp. 965–971, Feb. 2018, doi: 10.1021/acs.jpca.7b10299.

[5] S. Mukamel, *Principles of nonlinear optical spectroscopy* . in Oxford series in optical and imaging sciences ; 6. New York: Oxford University Press, 1995.

[6] Inc. Wolfram Research, “Mathematica, Version 13.1.” [Online]. Available: https://www.wolfram.com/mathematica

[7] A. B. Grafton *et al.*, “Excited-state vibration-polariton transitions and dynamics in nitroprusside,” *Nat Commun*, vol. 12, no. 1, p. 214, Jan. 2021, doi: 10.1038/s41467-020-20535-z.

[8] K. D. Rector, A. S. Kwok, C. Ferrante, A. Tokmakoff, C. W. Rella, and M. D. Fayer, “Vibrational anharmonicity and multilevel vibrational dephasing from vibrational echo beats,” *J Chem Phys*, vol. 106, no. 24, pp. 10027–10036, Jun. 1997, doi: 10.1063/1.474060.

[9] J. K. Vij, C. J. Reid, G. J. Evans, M. Ferrario, and M. W. Evans, “Molecular dynamics of CH2Cl2: temperature dependences of the far infra-red spectrum. Part 1: experimental and simulation,” *Advances in Molecular Relaxation and Interaction Processes*, vol. 22, no. 2, pp. 79–87, Feb. 1982, doi: 10.1016/0378-4487(82)80036-8.

[10] L. A. Blatz, “Low‐Frequency Raman Lines from Liquid Benzene, Benzene Derivatives, and Other Organic Liquids,” *J Chem Phys*, vol. 47, no. 2, pp. 841–849, Jul. 1967, doi: 10.1063/1.1711959.

[11] C. Stamper, D. Cortie, Z. Yue, X. Wang, and D. Yu, “Experimental Confirmation of the Universal Law for the Vibrational Density of States of Liquids,” *J Phys Chem Lett*, vol. 13, no. 13, pp. 3105–3111, Apr. 2022, doi: 10.1021/acs.jpclett.2c00297.

[12] A. Zaccone and M. Baggioli, “Universal law for the vibrational density of states of liquids,” *Proceedings of the National Academy of Sciences*, vol. 118, no. 5, Feb. 2021, doi: 10.1073/pnas.2022303118.

[13] M. Baggioli and A. Zaccone, “Explaining the specific heat of liquids based on instantaneous normal modes,” *Phys Rev E*, vol. 104, no. 1, p. 014103, Jul. 2021, doi: 10.1103/PhysRevE.104.014103.

[14] W. Schirmacher, T. Bryk, and G. Ruocco, “Comment on ‘Explaining the specific heat of liquids based on instantaneous normal modes,’” *Phys Rev E*, vol. 106, no. 6, p. 066101, Dec. 2022, doi: 10.1103/PhysRevE.106.066101.

[15] B. Xiang *et al.*, “Intermolecular vibrational energy transfer enabled by microcavity strong light–matter coupling,” *Science (1979)*, vol. 368, no. 6491, pp. 665–667, May 2020, doi: 10.1126/science.aba3544.

[16] A. Tokmakoff, B. Sauter, A. S. Kwok, and M. D. Fayer, “Phonon-induced scattering between vibrations and multiphoton vibrational up-pumping in liquid solution,” *Chem Phys Lett*, vol. 221, no. 5–6, pp. 412–418, Apr. 1994, doi: 10.1016/0009-2614(94)00276-2.

[17] H. Jobic and H. J. Lauter, “Calculation of the effect of the Debye–Waller factor on the intensities of molecular modes measured by neutron inelastic scattering. Application to hexamethylenetetramine,” *J Chem Phys*, vol. 88, no. 9, pp. 5450–5456, May 1988, doi: 10.1063/1.454555.

[18] S. N. Taraskin and S. R. Elliott, “Connection between the true vibrational density of states and that derived from inelastic neutron scattering,” *Phys Rev B*, vol. 55, no. 1, pp. 117–123, Jan. 1997, doi: 10.1103/PhysRevB.55.117.
